# An emulsion electrospun nanofibrous scaffold loaded with glial cell line-derived neurotrophic factor for nerve regeneration

**DOI:** 10.3389/fcell.2025.1567654

**Published:** 2025-04-16

**Authors:** Holly N. Gregory, Louis D. V. Johnson, James B. Phillips

**Affiliations:** ^1^ Department of Pharmacology, UCL School of Pharmacy, London, United Kingdom; ^2^ UCL Centre for Nerve Engineering, London, United Kingdom; ^3^ School of Chemical, Materials and Biological Engineering, University of Sheffield, Sheffield, United Kingdom

**Keywords:** electrospinning, nanofibres, nerve regeneration, growth factor, alignment, GDNF, PCL, Schwann cells

## Abstract

**Introduction:**

Damage to peripheral nerves is common in major trauma cases, and current options for surgical repair are often not sufficient to promote satisfactory recovery of sensory and motor function. In this study we describe the development of a biomaterial scaffold with aligned nanofibrous topography and encapsulated neurotrophic factor, designed to direct and enhance axonal regeneration and so effectuate faster return of function.

**Methods:**

Glial cell line-derived neurotrophic factor (GDNF) was loaded into aligned polycaprolactone (PCL) nanofibres using emulsion electrospinning, and the biomaterial was characterised alongside random and aligned PCL scaffolds without growth factor.

**Results and discussion:**

This fabrication route produced fine and uniform nanofibres with sustained release of GDNF over at least four weeks, and the aligned topography was able to orientate the growth of Schwann cells. Finally, the GDNF-loaded aligned nanofibrous scaffold significantly enhanced and directed the outgrowth of primary rat neurons cultured on its surface, demonstrating its promise as a pro-regenerative biomaterial for the surgical repair of nerve injury.

## Introduction

Peripheral nerves can be damaged through traumatic injury, for example in vehicular accidents or lacerations from sharp objects, and can also be affected by disease or surgical procedures (Murphy et al., 2023). Nerve injury often creates a significant deficit in motor and sensory function, and coupled with neuropathic pain these factors culminate in a marked reduction in patient quality of life (Heary et al., 2022; Felder and Ducic, 2021). Although peripheral nerves can regenerate after injury, cases of severe damage are often treated surgically in order to provide the structural continuity needed for regrowing axons to reach target tissues and restore function. These interventions commonly produce unsatisfactory outcomes for patients, in part due to the regenerative capacity of both axons (Sulaiman and Gordon, 2013) and Schwann cells (Jessen and Mirsky, 2019) diminishing over time. Further, functional recovery can be hindered by the misdirection of axons that do regenerate (Alant et al., 2013). Therapies that reinforce these regenerative processes and effectively guide axonal outgrowth would be valuable additions to the clinic to improve outcomes of surgical nerve repair.

Glial cell line-derived neurotrophic factor (GDNF) is a growth factor that potently stimulates neuronal outgrowth (Labroo et al., 2017; Lackington et al., 2019; Zhang et al., 2009; Santos et al., 2016) and survival (Henderson et al., 1994). After nerve injury, GDNF is highly upregulated by Schwann cells through the activation of protein kinase C/protein kinase D (Xu et al., 2013). However, this overexpression is short-lived, and its decline is associated with the eventual loss of regenerative capacity in axons (Hoke et al., 2002; Eggers et al., 2010). Controlled delivery of exogeneous GDNF to the site of nerve injury may resolve this limitation of the natural regeneration process, and allow robust recovery in scenarios of major nerve trauma (Eggers et al., 2019; Fadia et al., 2020).

Encapsulation of GDNF into a degradable polymeric scaffold, designed for implantation during nerve repair, may be particularly beneficial for this application through the temporary provision of guidance cues and mechanical support for regenerating tissue. The fabrication of aligned nanofibrous biomaterials using electrospinning has become a popular approach in preclinical nerve repair research, in part through their capacity to support and guide the outgrowth of neurons and Schwann cells (Xiong et al., 2023; Taylor et al., 2023) and positively modulate macrophage polarisation (Sun et al., 2024). Materials generated with this production method are well positioned for clinical translation, as the process is scalable and suitable for Good Manufacturing Practice requirements (Dziemidowicz et al., 2021). Electrospinning also permits the encapsulation of growth factors, and this can be achieved by processing a water-in-oil emulsion with polymer in organic phase and the therapeutic protein in aqueous phase using a surfactant. This procedure can minimise exposure of the protein to harsh solvent conditions while employing a relatively simple monoaxial instrument configuration, and studies using this technique have demonstrated bioactive growth factor release for nerve regeneration *in vitro* (Liu et al., 2021) and *in vivo* (Xia and Lv, 2018).

This study reports the development and characterisation of a novel biomaterial comprised of aligned GDNF-loaded polycaprolactone (PCL) nanofibres, manufactured via emulsion electrospinning. PCL is an FDA-approved polyester that is widely used in the fabrication of nerve repair scaffolds preclinically (Gregory and Phillips, 2021), and has a slow degradation rate (Dias et al., 2022) that may be beneficial in the repair of severe nerve damage where regeneration timeframes are extended. We first investigated nanofibre morphology and Schwann cell guidance capacity in the absence of growth factor, and then assessed the GDNF-loaded scaffold characteristics including growth factor release rate and structural and elemental composition. Finally, we explored the effect of GDNF delivery and aligned topography on the outgrowth of rat sensory neurons cultured on the scaffolds, in order to evaluate their potential to promote nerve regeneration.

## Materials and methods

### Nanofibre fabrication

#### Emulsion preparation

To a 2.5 mL solution of 12% 80 kDa polycaprolactone (PCL) in trifluoroethanol with 1% Span80, 250 µL of 5 mg/mL bovine serum albumin (BSA) in phosphate-buffered saline (PBS) containing 10 µg recombinant human GDNF was added dropwise over 2 min, with magnetic stirring at 300 RPM. The emulsion was then stirred at 900 RPM for 30 min and used for electrospinning immediately. For control blank nanofibres, GDNF was omitted from the aqueous protein solution.

#### Electrospinning

Electrospinning was carried out using a Fluidnatek LE-50 instrument (Bioinicia, Spain) set to 23°C and 35% humidity. Emulsion solutions were loaded into a 3 mL syringe and fed to a monoaxial spinneret fitted with a 1.4 mm (internal diameter) needle at 500 μL/h. A 100 mm-diameter mandrel covered with baking paper and positioned 20 cm from the spinneret was set to rotate at 1,750 RPM for the collection of aligned nanofibres, and 200 RPM for randomly orientated nanofibres. To generate more highly aligned materials, nanofibres were also collected on a disk rotating at 1,750 RPM (see Supplementary Material). All electrospun materials were generated under a voltage of 16.5 kV at the spinneret and −2.0 kV at the mandrel, then stored at −20°C until further characterisation. The emulsion formulation and collection conditions were selected to yield fine and uniform nanofibres with aligned architecture, based on optimisation performed in previous studies (Gregory, 2023).

#### Characterisation of external fibre morphology

Scanning electron microscopy (SEM) was used to investigate nanofibre morphology. Samples of each electrospun mat were arranged on a carbon tab and then sputter-coated with gold for 60 s using a Q150R coater (Quorum, United Kingdom). Nanofibres were imaged using a field emission Quanta 200 SEM (FEI, United Kingdom) alongside an Everheart-Thornley secondary electron detector. Fibre diameter was determined using manual measurement of at least 100 nanofibres per formulation across three images in ImageJ (Schneider et al., 2012). Fibre alignment was analysed with the Directionality plugin from ImageJ, using three images per formulation and setting the highest frequency to 0° to allow comparison between images acquired at different angles.

#### Characterisation of internal fibre morphology

Transmission electron microscopy (TEM) and energy-dispersive X-ray spectroscopy (EDS) were performed to assess the elemental composition and any internal structure of the nanofibres. Fibres were electrospun onto carbon/formvar-coated copper grids and imaged using a JEM-2100 TEM (JEOL, United Kingdom) under an accelerating voltage of 200 kV. EDS imaging was carried out with a mapping resolution of 512 × 512 pixels using Aztec software focussed on the detection of carbon, oxygen, nitrogen, sodium and chlorine.

#### GDNF release

To investigate the profile of GDNF release from the nanofibres, approximately 10 mg of fibres (in triplicate) were added to microtubes with 1 mL of PBS and heated at 37°C in an Incu-shake MINI shaking incubator (SciQuip, United Kingdom) with agitation at 75 RPM. At regular intervals the PBS was removed, stored at −20°C, and replaced with fresh. A GDNF ELISA kit was used to determine the concentration of GDNF in these samples. Briefly, standards (50 µL) or samples (40 µL) were combined with biotinylated antibody (10 μL, samples only) and streptavidin-horseradish peroxidase (50 µL) in a 96 well plate coated with GDNF antibody, and incubated for 60 min at 37°C. The plate was washed and two colorimetric detection substrates (50 µL each) introduced before a second incubation for 10 min, whereafter acidic stop solution (50 µL) was added and the absorbance was read at 450 nm using a Spectramax M2e microplate reader (Molecular Devices, United Kingdom).

#### Schwann cell culture on electrospun fibres

The SCL 4.1/F7 Schwann cell line (93031204) was acquired from the European Collection of Authenticated Cell Cultures, and maintained at subconfluence in high glucose Dulbecco’s Modified Eagle’s Medium (DMEM) with 10% fetal bovine serum (FBS), 100 U/mL penicillin, and 100 μg/mL streptomycin (P/S). Cells were detached from the culture surface using 0.25% trypsin/EDTA, then seeded at 25,000 cells/sample on 1 cm^2^ sections of nanofibre mat (random PCL and aligned PCL) that had been sterilised under UV and soaked in FBS for 15 min. Cultures were incubated for 48 h prior to fixation in 4% paraformaldehyde for 30 min at room temperature, ahead of immunocytochemistry. Experiments were conducted three times using three separate nanofibre squares per condition for each repeat.

#### Dissection and culture of primary rat neurons on electrospun fibres

Dorsal root ganglia were obtained from three adult Sprague-Dawley rats (two female and one male). Ganglia were dissected from the spinal column then incubated at 37°C in 0.125% collagenase type IV in DMEM with P/S for 90 min. The mixture was triturated to yield a dissociated cell suspension, then spin washed twice in supplemented DMEM before seeding into a T75 flask coated with 0.1% poly-d-lysine in the presence of 0.02 mM cytosine arabinofuranoside. Cultures of primary neurons were incubated overnight then detached with 0.25% trypsin/EDTA and resuspended in neurobasal A medium supplemented with 1% B-27, 2 mM l-glutamine and P/S. Neurons were seeded onto 1 cm^2^ sections of nanofibre mat (random PCL, aligned PCL, aligned GDNF, prepared as above) and incubated for 72 h before fixation in 4% paraformaldehyde for 30 min at room temperature. Experiments were performed three times, using three separate nanofibre squares per condition and tissue from one rat for each repeat.

#### Fluorescent labelling of cells on electrospun fibres

Nanofibre squares with cultured cells were first submerged in 0.1% Sudan black B in 70% ethanol for 40 min, followed by 0.5% Triton X-100 in PBS for 20 min. Those laden with Schwann cells were then immersed overnight in 1:200 rhodamine-conjugated phalloidin (00032, Biotium) in PBS at 4°C. Nanofibres with cultured primary neurons were treated with 5% goat serum in PBS followed by 1:500 Alexa Fluor® 488-conjugated anti-βIII tubulin (ab195879, abcam) in 1% goat serum in PBS overnight at 4°C. Thereafter all cells were exposed to 1:500 Hoechst 33258 in PBS for 10 minutes, and mounted using VECTASHIELD Vibrance® Antifade Mounting Medium (H-1700, Vector Laboratories).

#### Imaging and analysis

Primary neurons and Schwann cells cultured on electrospun nanofibres were imaged using a ×10 objective on an Axio Lab.A1 fluorescence microscope (Zeiss, Germany). For Schwann cells, three images were acquired at this magnification for each nanofibre square, and automated analysis on Volocity 6.5.1. software was employed to determine the angle of the cells in each field. These values were used to obtain the mean angle of each image, whereafter the degree of deviation for each individual cell was calculated. For primary neuron cultures, three distinct areas of the sample were visualised by stitching multiple images together to ensure these larger cells and their extending projections were in frame. Volocity was used to manually measure the length and prevailing angle of each object, and the longest five objects in each field were used in analysis of length and orientation. Samples were coded prior to imaging and analysis to avoid bias.

#### Statistics

Where applicable, statistical analyses were performed using GraphPad Prism 10.2.0. and the threshold for statistical significance established as P < 0.05. The following notation is used: ***P < 0.001, ****P < 0.0001.

## Results

**FIGURE 1:**
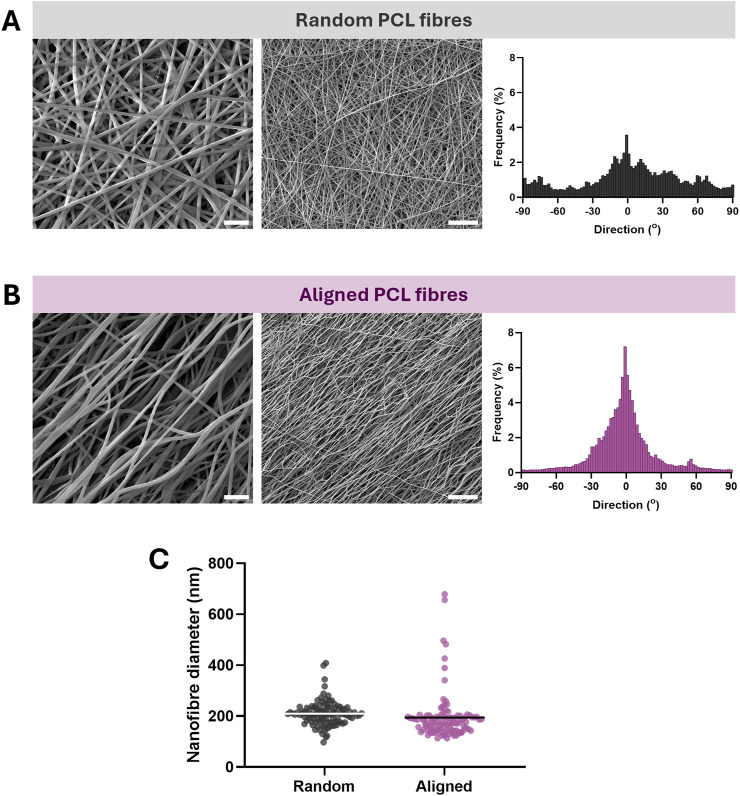
Electrospinning of emulsions generated from PCL and BSA produces nanofibres with uniform morphology, which align when collected at high mandrel speed. **(A)** SEM images of nanofibres collected at low mandrel speed (scale bar = 2 µm left, ×20,000 magnification, 10 µm right, ×4,000 magnification) and alignment frequency. **(B)** SEM images of nanofibres collected at high mandrel speed (scale bar = 2 µm left, ×20,000 magnification, 10 µm right, ×4,000 magnification) and greater frequency of aligned fibres. **(C)** Nanofibre diameter of random and aligned materials from at least 100 points of measurement per formulation, line = mean.

Our initial experiments loaded the carrier protein BSA into the nanofibres, to assess formulation suitability prior to the encapsulation of growth factor. Electrospinning of BSA-loaded PCL emulsions with collection onto a mandrel rotating at 200 RPM resulted in the production of discrete and consistent nanofibres, with randomly orientated morphology (Figure 1A). Increasing the mandrel rotation speed to 1,750 RPM effectively aligned the nanofibres in parallel with the direction of rotation, and maintained uniformity in fibre morphology (Figure 1B). In addition, using a 100 mm disk collector at 1,750 RPM in place of the mandrel generated nanofibres with more highly aligned morphology (Supplementary Figure S1A), although the yield using that production methodology was considerably lower. The average fibre diameter was comparable between the randomly orientated and aligned scaffolds, with mean diameters of 210 ± 48 nm (±SD) and 194 ± 92 nm (±SD) respectively (Figure 1C). Therefore, monoaxial emulsion electrospinning of protein-loaded solutions can be employed to fabricate aligned nanofibrous biomaterials with low diameter.

**FIGURE 2:**
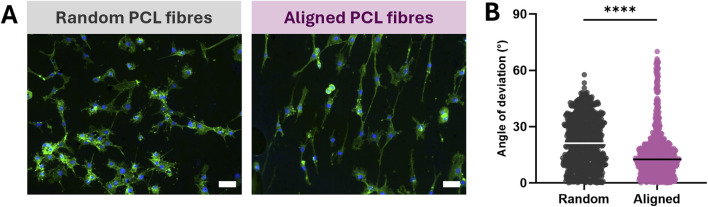
Schwann cells cultured on aligned emulsion electrospun nanofibres display orientated morphology. **(A)** Fluorescence images of Schwann cells on random and aligned nanofibres (scale bar = 50 µm) stained with phalloidin (green) and Hoechst (blue), acquired at ×10 magnification. **(B)** Orientation of Schwann cells determined by the angle of deviation from the mean angle of each image, line = mean. N = 9 individual nanofibre samples per condition over three experimental repeats, using an independent culture of Schwann cells for each repeat, unpaired two-tailed T-test where ****P < 0.0001.

In order to assess whether nanofibre orientation could influence the morphology of cells cultured on the scaffold surface, we seeded Schwann cells onto random and aligned materials and quantified their directionality after 48 h in culture. Both materials supported robust cell growth, and those grown on aligned PCL nanofibres exhibited an elongated shape with extended projections and evident orientation of cells (Figure 2A). Conversely, Schwann cells cultured on random PCL nanofibres appeared less elongated and did not display obvious directionality. Nanofibres generated using the disk collector also appeared to orientate Schwann cell growth to an equivalent extent (Supplementary Figure S1B). Given that Schwann cell directionality was not greater on the disk nanofibres, and this method was less practicable for the fabrication of scaffolds than collection on the mandrel, materials generated using the disk collector were not investigated further. Analysis of individual cell angles revealed that Schwann cells on aligned PCL nanofibres had a significantly lower mean deviation (P < 0.0001) from the average orientation compared with those on random scaffolds, indicating more highly aligned cell morphology (Figure 2B). These data indicate that the aligned scaffolds can support and direct the growth of Schwann cells, a cell type critical in nerve regeneration.

**FIGURE 3:**
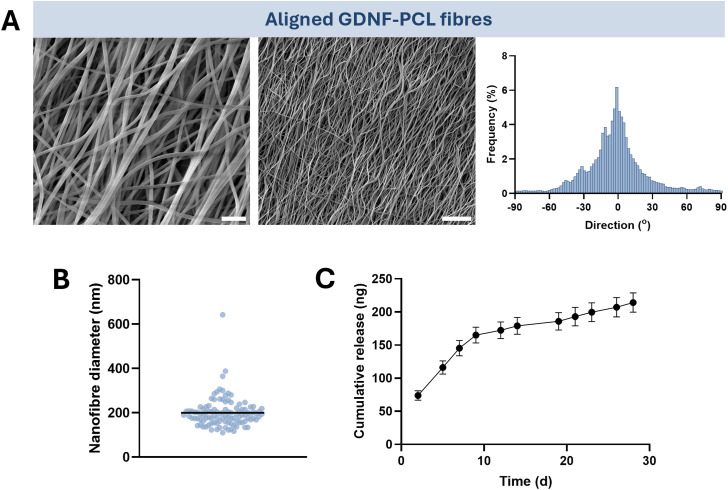
Emulsion electrospun nanofibres can be loaded with the growth factor GDNF, retain uniform morphology and alignment, and demonstrate sustained release of GDNF over 4 weeks. **(A)** SEM images of GDNF-loaded nanofibres collected at high mandrel speed (scale bar = 2 µm left, ×20,000 magnification, 10 µm right, ×4,000 magnification) and alignment frequency. **(B)** Nanofibre diameter of aligned material from at least 100 points of measurement, line = mean. **(C)** Cumulative release of GDNF from 10 mg of nanofibres over 28 days, mean ± SD. N = 3 individual nanofibre samples.

Having generated an aligned protein-loaded scaffold suitable for the guidance of Schwann cells, we used an identical formulation and collection conditions to encapsulate GDNF into emulsion electrospun nanofibres. This includes the loading of BSA, which was incorporated to protect the growth factor during electrospinning and after release from the biomaterial. Addition of GDNF to the emulsion was not overtly detrimental to the morphology of the nanofibres, which retained a smooth and linear appearance and an acceptable degree of alignment (Figure 3A). Similarly, the nanofibres had a low mean diameter of 200 ± 66 nm (±SD), which is highly comparable to those without encapsulated GDNF (Figure 3B). Lastly, we observed that release of the growth factor from the scaffold *in vitro* was sustained over at least 4 weeks, reaching a cumulative total of 214 ± 15 ng (±SD) at 28 days of release into PBS (Figure 3C). As such, aligned PCL nanofibres demonstrating sustained release of GDNF can be fabricated by emulsion electrospinning.

**FIGURE 4:**
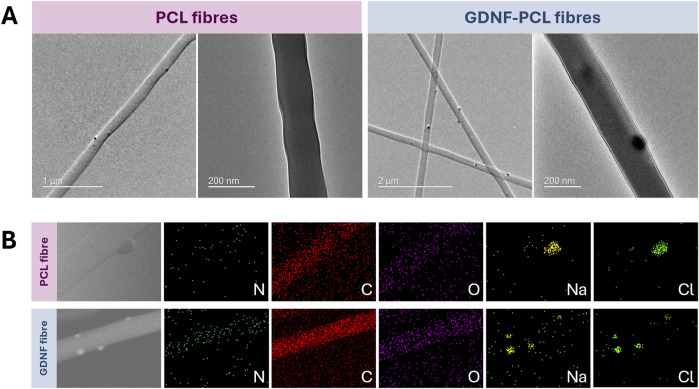
Structural and elemental characterisation of GDNF-PCL and PCL only nanofibres, using transmission electron microscopy (TEM) and energy dispersive X-ray spectroscopy (EDS). **(A)** TEM images of PCL only nanofibres (left) and GDNF-loaded nanofibres (right), displaying presence of electron-dense clusters on fibre surface. **(B)** EDS images of PCL only nanofibre (top) and GDNF-loaded nanofibre (bottom) displaying elemental composition of nitrogen, carbon, oxygen, sodium and chlorine.

Thereafter, we sought to interrogate the structure and elemental composition of the nanofibres, using TEM and EDS analyses. TEM imaging revealed the presence of small, relatively electron dense clusters on or near the nanofibre surface in PCL materials loaded with both GDNF and BSA, and those without any protein (Figure 4A). High magnification images did not demonstrate evidence of a clear core-shell structure, with both formulations appearing relatively homogeneous bar the presence of the surface aggregates. Elemental mapping of the scaffolds with EDS reflected the existence of carbon and oxygen within the nanofibres, and also notably detected nitrogen in the materials loaded with protein (Figure 4B). Interestingly, the surface clusters observed in TEM imaging appeared to correlate to the spatial distribution of sodium and chlorine in the EDS analysis, suggesting they are comprised at least in part of these elements. These characterisations offer insight into the internal structure of the nanofibres, and could suggest the successful encapsulation of protein.

**FIGURE 5:**
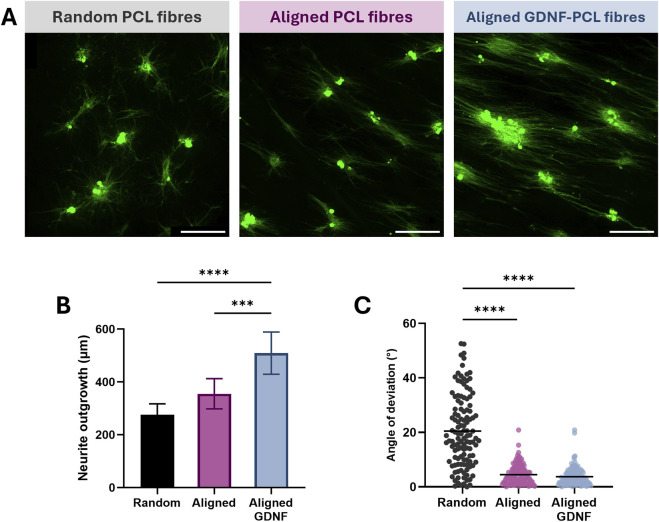
GDNF-loaded nanofibres enhance and direct outgrowth of neurites from primary rat sensory neurons cultured on their surface. **(A)** Fluorescence images of neurons from rat dorsal root ganglia cultured on random PCL (left) aligned PCL (centre) and GDNF-loaded aligned PCL nanofibres (right) (scale bar = 200 µm) immunostained for βIII-tubulin (green), acquired at ×10 magnification. **(B)** Outgrowth length of neurites from primary neurons on random, aligned and GDNF-loaded aligned nanofibres, mean ± SD. **(C)** Orientation of neurite outgrowth on nanofibres, line = mean. N = 9 individual nanofibre samples per condition over three experimental repeats, using an independent culture of primary neurons for each repeat, one-way ANOVA with Tukey’s post-hoc test where ****P < 0.0001 and ***P < 0.001.

Finally, we evaluated the potential of the aligned GDNF-loaded nanofibrous scaffold to enhance regeneration, by culturing sensory neurons from rat dorsal root ganglia on their surface. The degree and orientation of outgrowth was investigated after 3 days *in vitro*, comparing this substrate to random and aligned scaffolds without GDNF in order to isolate any influence of aligned topography vs. eluted neurotrophin. All biomaterials promoted robust cell attachment and outgrowth of processes, with both sets of aligned nanofibres stimulating directional growth of neurites (Figure 5A). Although neuronal cultures appeared more dense on GDNF-loaded materials, we did not quantify this formally. The GDNF-loaded scaffolds did significantly enhance neuronal outgrowth compared with both random and aligned controls, amounting to an increase of more than 80% in length over random materials (P < 0.0001) and in excess of 40% compared with aligned nanofibres that did not contain growth factor (P < 0.001) (Figure 5B). Although these aligned scaffolds themselves appeared to increase average neurite outgrowth compared with randomly orientated fibres, this difference was not statistically significant. Both aligned and aligned GDNF-loaded conditions produced a significant reduction in mean angle of deviation over random nanofibres (P < 0.0001), confirming their capacity to effectively direct the growth of neuronal processes (Figure 5C). In summary, the aligned GDNF-loaded PCL nanofibres presented here can promote orientated and accelerated outgrowth from primary rat neurons cultured on their surface, demonstrating their potential as scaffolds for nerve regeneration.

## Discussion

In this study we sought to fabricate a biomaterial for peripheral nerve repair that combined multiple attributes known to promote regeneration independently, with the particular objectives of directing and enhancing the regeneration of axons and encouraging the alignment of Schwann cells. Emulsion electrospinning was used to create nanofibrous PCL scaffolds with aligned topography and encapsulated GDNF, and to our knowledge this is the first report of this combination of pro-regenerative aspects fabricated by this route. This combinatorial approach could offer biological advantages over existing technologies that focus primarily on aligned architecture, and may be more straightforward to manufacture at scale compared to systems using a separate component (e.g., cells) to supply neurotrophic factors.

Our preliminary experiments developed the emulsion electrospinning process using the encapsulation of BSA within random and aligned PCL fibres, as this protein would also be present in the GDNF-loaded formulation to protect the growth factor. The addition of BSA in the electrospinning of growth factors is a common strategy (Hu et al., 2016; Kijeńska-Gawrońska et al., 2019; Liu et al., 2018a), based on its physiological function as a carrier for compounds in circulation (Spada et al., 2021) and ability to inhibit the misfolding and aggregation of other proteins (Finn et al., 2012). The resultant scaffolds from both processes were uniform with nanofibres around 200 nm in diameter, which is comparable to aligned PCL nanofibres loaded with nerve growth factor generated using a rotating mandrel, with an average diameter of 320 nm (Hu et al., 2016). In addition, aligned nanofibres were effectively generated here by increasing the rotational speed of the collecting mandrel – this effect is well documented and occurs through mechanical forces exerted on the fibre as it is drawn into the collector, that surpass the effect of the jet bending instability which would otherwise lead to random fibres (Robinson et al., 2021).

Although not measured in this work, it is also important for future clinical applications to evaluate batch-to-batch variability in the morphology of these nanofibrous scaffolds, and establish an acceptable range. Our previous work on electrospun aligned PCL nanofibres loaded with a small molecule indicated that fibre diameter varied by < 9% across three batches (Gregory et al., 2024), which speaks to the consistency of this production method.

The random and aligned materials were then used as scaffolds for the culture of Schwann cells, to assess whether the nanofibre surface could support and guide cell growth. Both materials were suitable as growth substrates, and the aligned nanofibres produced an orientated and elongated morphology in the cultured Schwann cells consistent with previous reports from our lab (Gregory et al., 2024) and others (Gnavi et al., 2015). During nerve regeneration, cords of orientated Schwann cells guide the growth of axons from the proximal to the distal stump (Standring, 2020), and so alignment of these cells on the surface of a repair biomaterial may aid in this process. In future studies it would be valuable to investigate Schwann cell behaviour on the nanofibres in greater detail, for example by measuring proliferation, migration, and viability.

Having developed a robust BSA-loaded biomaterial capable of supporting Schwann cell alignment *in vitro*, this formulation was used to fabricate aligned PCL nanofibres with encapsulated GDNF. Emulsion electrospinning has been used previously to load GDNF into polymeric nanofibres for nerve repair, using poly(lactic-co-glycolic) acid (Liu et al., 2018a; Liu et al., 2018b; Liu et al., 2021). These scaffolds exhibited sustained and bioactive growth factor release over 6 weeks, but degraded by 44% in the same period – this timeframe may be too rapid compared to the rate of axonal regeneration in humans, where severe nerve injuries would likely benefit from regenerative support for weeks to months. These studies motivated our choice of PCL, given its slower rate of degradation. Electrospun GDNF-loaded fibres have been reported using PCL (Mohtaram et al., 2015), in addition to a caprolactone-ethyl ethylene phosphate (PCLEEP) copolymer (Chew et al., 2007). These reports appear to employ blend electrospinning, involving addition of the growth factor (and protectant BSA) directly to a solution of polymer and organic solvent. Here we present an alternative approach using emulsion electrospinning: this technique introduces a surfactant to stabilise the organic and aqueous mixture, and in PCL fibres has been demonstrated to produce more controlled drug release than blend electrospinning (Hu et al., 2015; Radisavljevic et al., 2018).

We assessed morphology of the GDNF-loaded nanofibres, which was highly comparable to previous scaffolds loaded only with BSA, and evaluated the magnitude and duration of GDNF release, which was sustained over at least 28 days. It would be beneficial to establish the kinetics of release over a longer timeframe, in order to determine whether this scaffold is capable of providing long-term neurotrophic support to regenerating axons. Importantly, we observed that approximately 200 ng of growth factor was eluted from 10 mg of biomaterial over this 4-week period. In a study evaluating the efficacy of PCL nerve guides containing GDNF-loaded microspheres used to repair 5.0 cm median nerve gaps in non-human primates, the authors describe that 14 ng of growth factor was released from the materials over 50 days (Fadia et al., 2020). Similarly, in what may be the sole account of GDNF-loaded electrospun fibres tested in an animal model, the researchers reporting the GDNF-PCLEEP fibres in the repair of rat sciatic nerve note that 17.7 ng of the therapeutic protein was released from their constructs over 3 months (Chew et al., 2007). Accordingly, for any future *in vivo* assessment of the present technology, the mass of biomaterial used for repair should be carefully considered in order to provide a physiologically effective dose, and prevent instances of axonal trapping that can occur under excessive local GDNF concentrations (Eggers et al., 2013).

It will be necessary in future development of this biomaterial to investigate the total mass of GDNF encapsulated during manufacture, and the reproducibility of this aspect. We expect some minor loss of protein in electrospinning compared to that theorectically loaded, with the encapsulation efficiency of GDNF reported to be around 80% in similar systems (Liu et al., 2018b). The electrospinning instrument used in this study allows for precise control over day-to-day fluctuations such as temperature and humidity, which may mitigate the influence that these factors can exert over nanofibre properties and so minimise variability (Ramazani and Karimi, 2014; Nezarati et al., 2013).

Thereafter, TEM and EDS were used to interrogate the structural and elemental composition of the GDNF nanofibrous scaffolds, and the images compared to PCL nanofibres without loaded protein. The GDNF-loaded fibres did not exhibit a biphasic core-shell structure, which has been observed for other emulsion electrospun PCL nanofibres and involves the protein residing in a continuous core within the fibre (Norouzi et al., 2022). Our use of PBS during emulsion preparation may have provided ions that impacted stability and prevented the formation of this structure (Wang et al., 2014), however this lack of obvious internal core is not a barrier to its application. TEM of both GDNF-PCL and PCL scaffolds revealed dark clusters on the fibres, and EDS established that these areas were positive for both sodium and chlorine. These likely originated from PBS, and we hypothesise that the application of high voltage during electrospinning resulted in the migration and clustering of these ions at the nanofibre surface. The EDS also confirmed the presence of nitrogen only in the scaffolds loaded with protein, and these results echo similar analyses of electrospun poly(l-lactide)-caprolactone fibres loaded with brain-derived neurotrophic factor (Ni et al., 2024).

Finally, primary rat sensory neurons were cultured on the random, aligned, and aligned GDNF-loaded scaffolds produced in this study. We determined that the aligned nanofibrous topography could influence the directionality of neurite outgrowth, an effect that is observed in both sensory and motor neurons (Corey et al., 2008). Further, GDNF released from the scaffold could greatly increase the length of these directed neurites. GDNF encourages neurite outgrowth in dissociated cultures of adult rat sensory neurons via RET/PI3K pathway signalling, and has been reported to produce an almost two-fold increase in neurite length in these cells (Takaku et al., 2013). We observed a similar effect size between random and GDNF-loaded aligned scaffolds, suggesting that the neurotrophic factor remained bioactive after the electrospinning process and was released *in vitro* at a therapeutic concentration.

Future development of this technology should test the capacity of this aligned nanofibrous biomaterial to improve outcomes of nerve repair *in vivo*, in order to gain a fuller picture of its influence alongside the complex signalling cascades and numerous cell types involved in regeneration. Initially, this would require studies addressing the challenge of tuning the timing, duration and concentration of GDNF release to promote axonal regeneration without entrapment (Eggers et al., 2020).

In this study we have presented an electrospun scaffold with encapsulated neurotrophic factor and aligned topography for nerve regeneration. Taken with our recent publication reporting the loading of a small molecule therapeutic into aligned fibres (Gregory et al., 2024), we have assembled a flexible delivery platform that could potentially incorporate a wide range of proteins and drugs to advance clinical options for the repair of severe nerve injury.

## Data Availability

The raw data supporting the conclusions of this article will be made available by the authors, without undue reservation.
