# Supplementary material for: An emulsion electrospun nanofibrous scaffold loaded with glial cell line-derived neurotrophic factor for nerve regeneration
Source: Front Cell Dev Biol. 2025 Apr 16;13:1567654. doi: 10.3389/fcell.2025.1567654 (PMC12040939; doi:10.3389/fcell.2025.1567654)
Supplement: Supplementary file 1 [file DataSheet1.docx]

## **Supplementary information**

### **Methods**

#### **Preparation of emulsion electrospun fibres using disk collector**

Solutions of BSA in PBS and PCL in TFE were prepared and combined using the same method as previously to form an emulsion, without the addition of GDNF. Nanofibres were generated under identical conditions, except in collection of the fibres the 100 mm diameter mandrel was replaced with a 100 mm diameter disk rotating at 1750 RPM, and baking paper was not used as a collection substrate. Nanofibre orientation and diameter were determined from SEM images in the same manner as previous formulations.

#### **Schwann cell culture on disk aligned nanofibres**

Schwann cells were cultured on 1 cm^2^ samples of aligned nanofibres manufactured using the disk collector using the same protocol as was employed for random and aligned nanofibres generated using the mandrel collector. Cells were stained for F-actin as previously and their orientation quantified from fluorescence images.


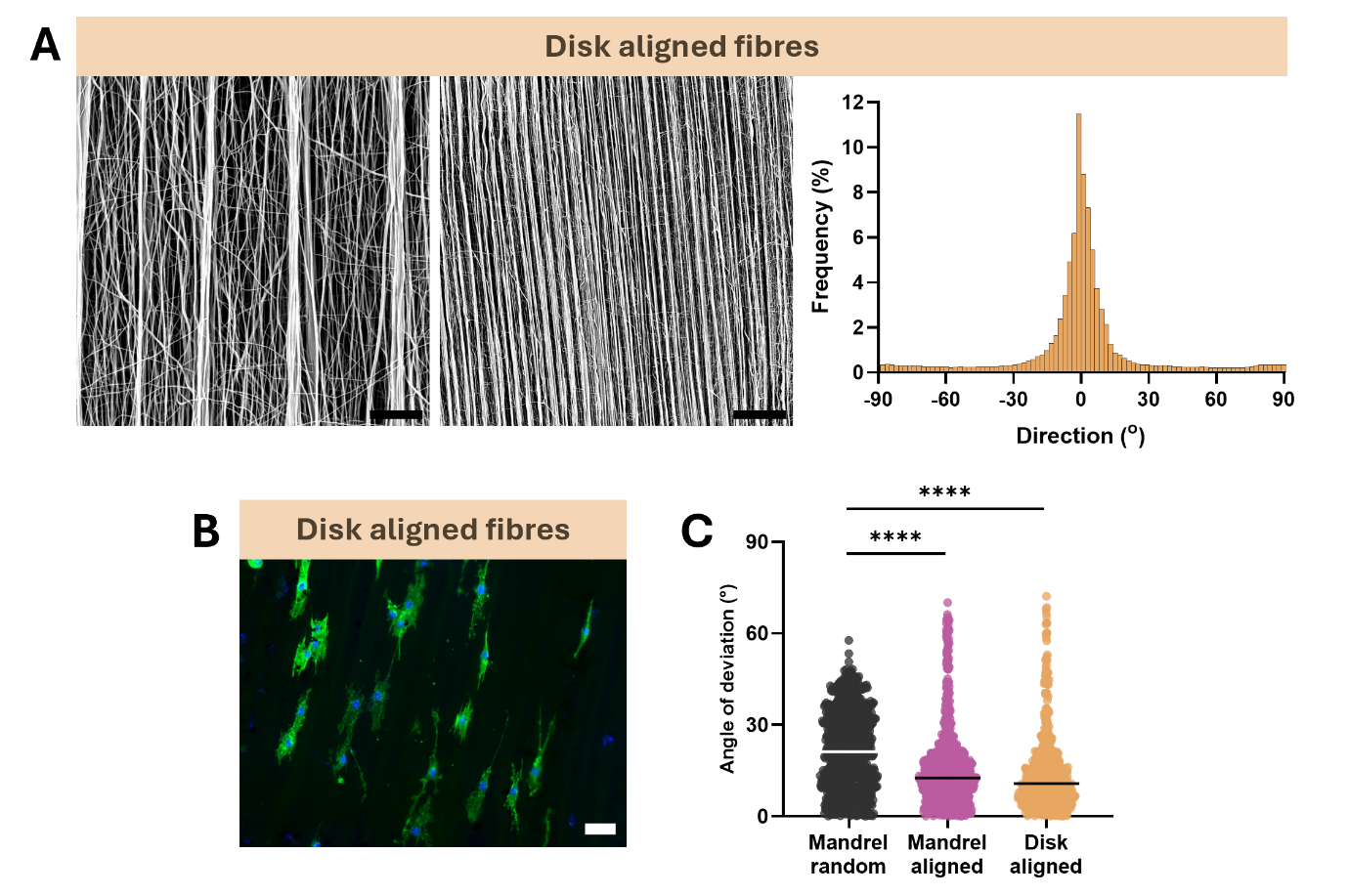


**Supplementary Figure 1: Emulsion electrospun nanofibres collected on a rotating disk display highly aligned morphology and promote equivalent orientation of Schwann cells to those collected on a rotating mandrel.** **A** SEM images of nanofibres collected at high disk collector speed (scale bar = 10 µm left, X4,000 magnification, 40 µm right, X1,000 magnification) and alignment frequency. **B** Fluorescence images of Schwann cells on random and aligned nanofibres (scale bar = 50 µm) stained with phalloidin (green) and Hoechst (blue), acquired at X10 magnification. **C** Orientation of Schwann cells determined by the angle of deviation from the mean angle of each image, line = mean. N = 9 individual nanofibre samples per condition over three experimental repeats, using an independent culture of Schwann cells for each repeat, one-way ANOVA with Tukey’s post-hoc test where **** P<0.0001.
